# Signet Ring Cell Carcinoma Presenting as a Necrotic Duodenocolic Fistulizing Mass

**DOI:** 10.7759/cureus.38361

**Published:** 2023-04-30

**Authors:** Shiva F Naidoo, Hamzah Shariff, Dhruv Lowe, Abhinav Goyal, Divey Manocha

**Affiliations:** 1 Internal Medicine, Geisinger Health System, Wilkes-Barre, USA; 2 Medical Education, Geisinger Commonwealth School of Medicine, Scranton, USA; 3 Gastroenterology and Hepatology, Geisinger Health System, Scranton, USA; 4 Gastroenterology, Geisinger Health System, Scranton, USA

**Keywords:** duodenal fistula, git endoscopy, signet ring cell adenocarcinoma, bowel fistula, colorectal cancer

## Abstract

A 79-year-old female was referred for endoscopic evaluation after a positive fecal occult blood test. There was a long-standing history of iron deficiency anemia, weight loss with intermittent touts of intractable vomiting, and nausea. Esophagogastroduodenoscopy revealed a secondary lumen between the duodenum and transverse colon with necrotic mucosa and a blind opening. Subsequent colonoscopy revealed similar necrotic mucosa at the transverse colon and fistula formation with communication into the duodenum. Signet ring cell carcinoma (SRCC) was evident in histologic analysis. SRCC carries a poorer prognosis than other variants of colorectal carcinoma (CRC). Proposed mechanisms of increased mucin production can lead to mucosal wall destruction and have profound manifestations, such as in our patient with duodenocolic fistula.

## Introduction

Signet ring cell carcinoma (SRCC) is a rare form of colorectal carcinoma (CRC) associated with a poor prognosis and higher mortality. SRCC histologically are characterized by cells that produce mucin that displaces the nucleus to the periphery of the cytoplasm, which resembles a signet ring. Increased mucin production facilitates further destruction of colonic mucosa, contributing to the aggressive nature of this neoplasm [1]. 

Colonic fistula formation is a rare condition, but most associated with Crohn’s disease. SRCC is a rare etiology of fistulation. Frequent injury to the colonic mucosa can lead to inflammation and remodeling. Subsequently, ulceration of the wall may invade adjacent structures, such as other parts of the bowel or organs. Most cases in the literature have both a history of inflammatory bowel disease and SRCC. It is even rarer to find such a presentation with SRCC alone. 

## Case presentation

A 79-year-old female was referred for endoscopic evaluation by her primary care physician for a positive fecal occult blood test, along with evidence of iron deficiency anemia. Past medical history was notable for a cerebrovascular accident/ischemic stroke, vascular dementia, morbid obesity, tobacco abuse, and type 2 diabetes mellitus. The patient had a previous history of intermittent vomiting and nausea, which resolved with medical therapy over the last four years. However, in the last five months, her vomiting episodes persisted. There were no complaints of hematemesis, and abdominal pain as well. 

Subsequently, there was a reported 32-pound weight loss in the last seven months. There was no prior history or family history of any malignancy, intraabdominal surgery, or inflammatory bowel disease. 

Prior serologic workup was notable for a hemoglobin of 8.5 g/dL, which has been on a decline from two years prior, and previous baseline hemoglobin of around 14.0 g/dL. Iron studies revealed an iron level of 19, a transferrin binding capacity of 213, and a transferrin saturation percent of 9. The rest of the initial serology was unremarkable. 

**Figure 1:**
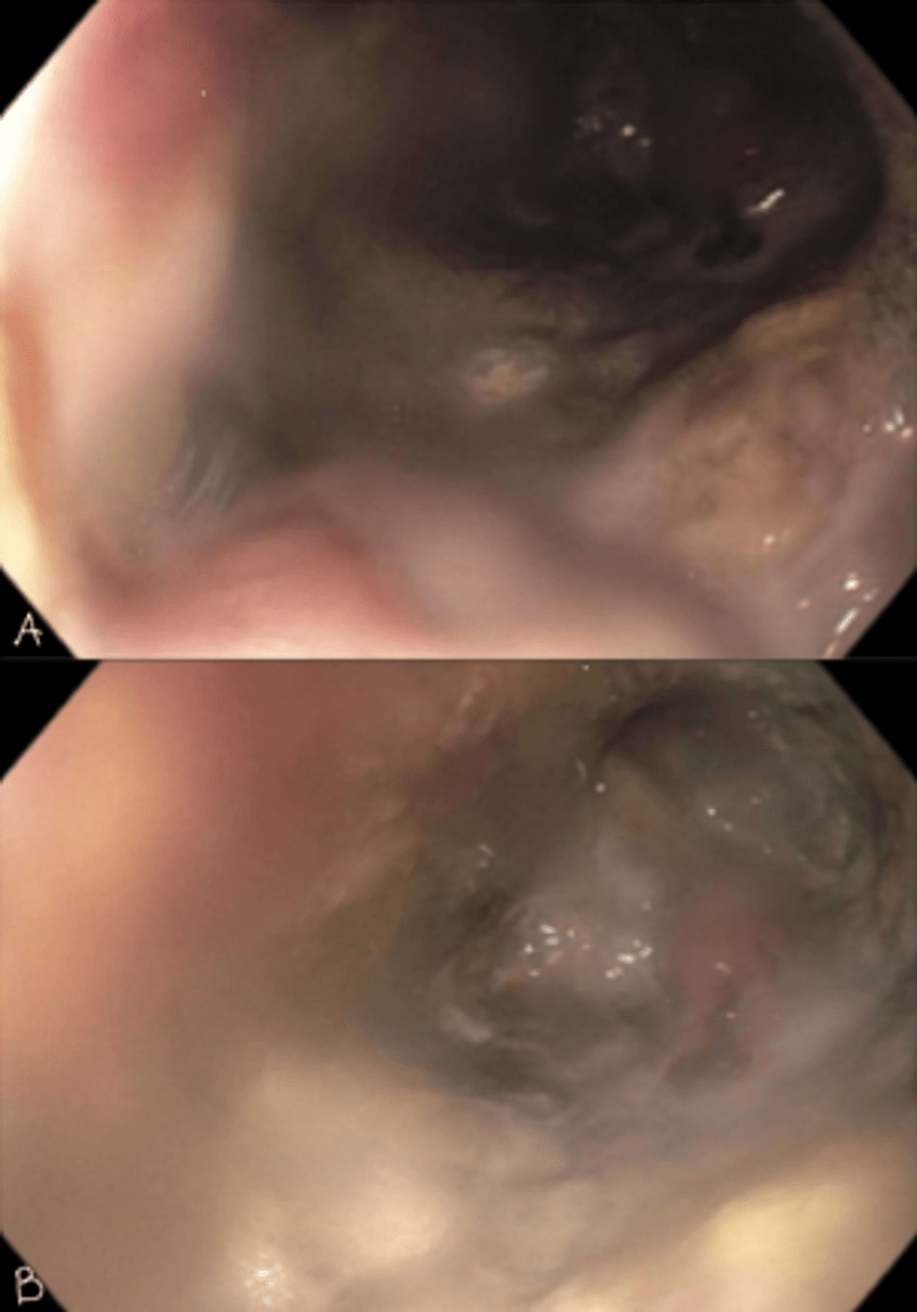
EGD most notable for necrotic mucosa and secondary lumen with blind pouch fistula. A. Second part of duodenum, B. Invagination from duodenum into duodenocolic fistula and necrotic mass

Esophagogastroduodenoscopy (EGD) performed initially revealed a necrotic mucosal outpouching with a secondary lumen in the second part of the duodenum. Upon advancement into the secondary lumen, necrotic mucosa and an open-ended secondary lumen was noted (Figure 1). 

**Figure 2:**
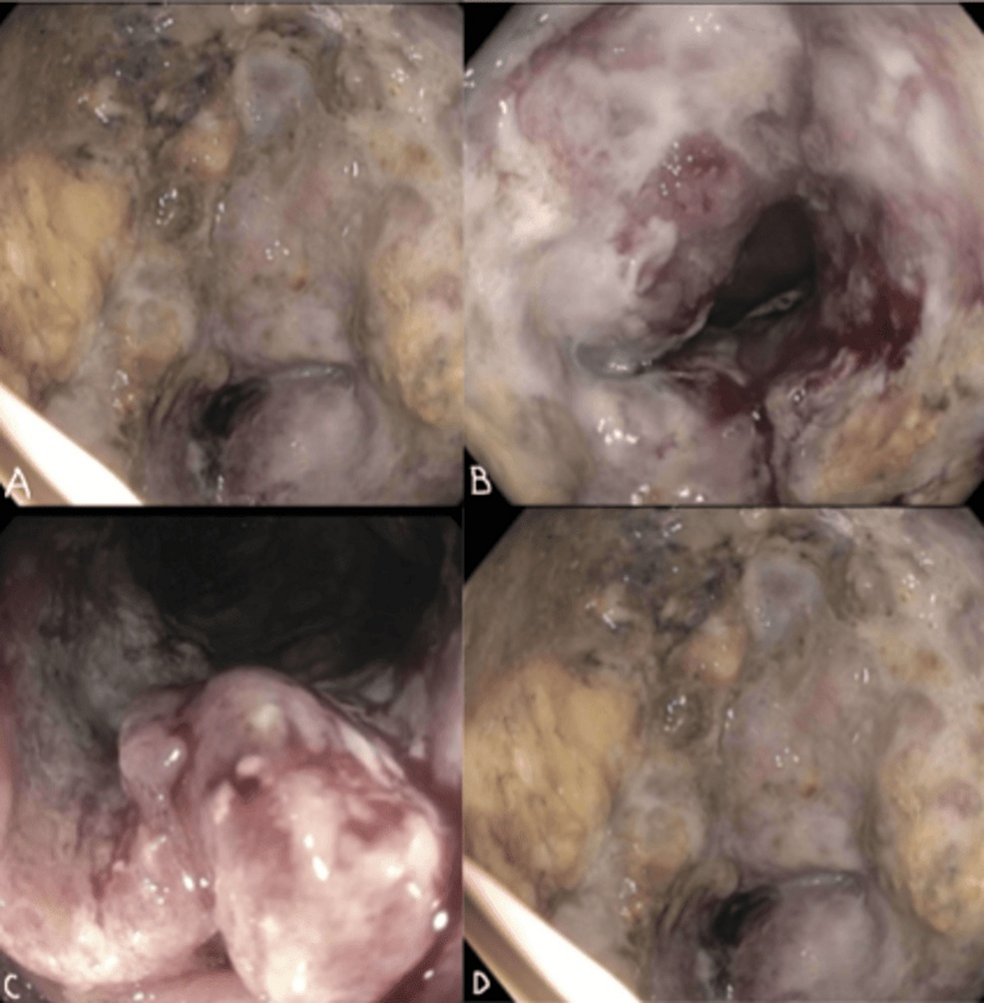
Colonoscopy images most notable at transverse colon 60 cm proximal to anus, with secondary lumen and fistula communicating into duodenum, with the same necrotic mucosa observed from EGD. A. Initial Proximal Transverse Colon, B. Subsequent Proximal Transverse Colon, C. Invagination of secondary duodenocolic fistula, D. Communication into duodenum from duodenocolic fistula with profound necrosis

Subsequent colonoscopy in the transverse colon revealed necrotic mucosa with a secondary lumen, similar to findings on EGD. The colonoscope was advanced through the secondary lumen, which communicated into the duodenum (Figure 2). 

Biopsies were obtained and the patient returned to her skilled nursing home. 

**Figure 3:**
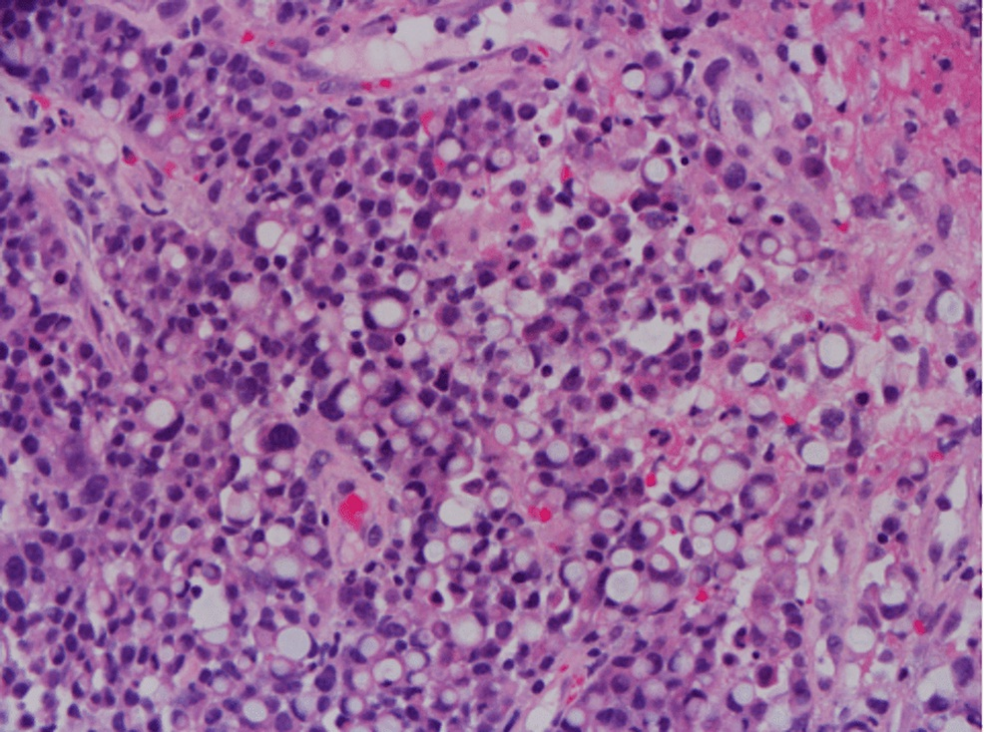
Poorly differentiated adenocarcinoma with solid sheets of cells, with focal signet ring features.

Pathology resulted in both the duodenal and colon mass with evidence of invasive adenocarcinoma with focal signet ring features (Figure 3). Also of note, candida esophagitis and chronic gastritis with staining for H. pylori were negative. Genetic analysis was notable for a positive V600E/V600D mutation in BRAF Codon. MLH1, MSH-2, MSH-6, PMS2, KRAS codons, and NRAS codons were unremarkable. 

Computed tomography of the chest, abdomen, and pelvis was ordered for staging. However, with subsequent goals of care discussion, it was decided to proceed with palliative management. Subsequent workup was discontinued. 

## Discussion

CRC accounts for an estimated 7.9% of all new cancers and 8.6% of cancer deaths in the United States [2]. In recent years, the incidence and mortality of CRC have been declining, with further emphasis on screening and prevention. 

Our patient did not have CRC screening and presented with alarming symptoms that warranted urgent endoscopic evaluation. The importance of screening colonoscopies is paramount and was demonstrated in a systematic review of 7,713,778 individuals that showed a 54% relative risk (RR) reduction in incidence (RR: 0.48, 95% confidence interval (CI): 0.46-0.49) and 62% RR reduction in mortality (RR: 0.38, 95% CI: 0.36-0.40) respectively [3]. 

CRC is classified by the World Health Organization (WHO), as comprising adenocarcinoma, neuroendocrine tumor, neuroendocrine carcinoma, and mixed neuroendocrine-non-neuroendocrine neoplasm [4]. SRCC is a rare form of adenocarcinoma accounting for 0.1 to 2.4% of all CRCs. 

Histologic morphology grade has been shown to be an independent factor of staging in prognosis. SRCC is associated with a poorer prognosis. It is hypothesized that extracellular mucin can penetrate through colonic wall tissue, further extending tumor invasion. Signet ring cell features mucin that can be concentrated within the lumen [1]. SRCC histology was associated with a higher risk of death hazard ratio (HR) 1.42 (CI 1.33-1.55) in the colon [1]. 

Genetic markers such as BRAF, which are associated with smoking, are also associated with negative prognosis [5]. In our patient, the history of smoking was one of the few risk factors evident. 

Duodenocolic fistula formation is an even rare finding. Mostly associated with Crohn's disease, but other etiology includes tuberculosis, gallstone disease, and malignancy from the hepatic flexure [6].

Treatment of fistulas is mostly surgical intervention with possible en bloc duodenopancreatectomy, but nutritional support is key too [5]. 

## Conclusions

SRCC is a rare form of CRC that carries a poor prognosis. The aggressive nature of this neoplasm can have profound effects, such as the development of a duodenocolic fistula. Vigilant consideration of disease manifestation, may lead to earlier screening, and intervention to prevent significant complications that follows. 
